# Establishment of a clinically relevant beagle model for periprosthetic joint infection with 3D-printed prostheses and multimodal evaluation

**DOI:** 10.1016/j.jot.2025.05.007

**Published:** 2025-07-03

**Authors:** Heng Liu, Tingting Fan, Rui Yuan, Shuai Lu, Dadi Sun, Yong Huan, Maoqi Gong, Honghu Xiao, Chongbin Wei, Hao Wang, Shijie Fan, Yilong He, Jialin Hu, Haoran Zhang, Hao Sun, Qi Gu, Yejun Zha, Xieyuan Jiang

**Affiliations:** a Department of Orthopedic Trauma, Beijing Jishuitan Hospital, Capital Medical University, Beijing, 100035, China; b State Key Laboratory of Organ Regeneration and Reconstruction, Institute of Zoology, Chinese Academy of Sciences, Beijing, 100101, China; c University of Chinese Academy of Sciences, Beijing, 100049, China; d Beijing Research Institute of Traumatology and Orthopaedics, Beijing, 100035, China; e Department of Foot and Ankle Surgery, Beijing Tongren Hospital, Capital Medical University, Beijing, 100730, China; f Beijing Institute for Stem Cell and Regenerative Medicine, Beijing, 100101, China; g State Key Laboratory of Nonlinear Mechanics (LNM), Institute of Mechanics, Chinese Academy of Sciences, Beijing, 100190, China; h School of Engineering Science, University of Chinese Academy of Sciences, Beijing, 100049, China; i Beijing AK Medical Co., Ltd., Beijing, 102200, China; j Department of Orthopedics, Beijing Jishuitan Hospital Guizhou Hospital, Guizhou, 550014, China; k Peking University Fourth School of Clinical Medicine, Beijing, 100035, China

**Keywords:** Periprosthetic joint infection (PJI), 3D printed prosthesis, Beagle model, Translational biomechanics

## Abstract

**Objective:**

Periprosthetic joint infection (PJI) poses significant challenges to arthroplasty outcomes, necessitating translational animal models for pathogenesis studies and therapeutic development. This study aimed to establish a standardized Beagle PJI model by integrating species-specific 3D-printed femoral prostheses with quantitative bacterial inoculation, while evaluating the dose-dependent effects of *Staphylococcus aureus* (*S. aureus*) on infection progression.

**Methods:**

Two titanium alloy prostheses were designed using CT-based anatomical data: BFP-C (canine-optimized) and BFP-H (human-derived). Prostheses underwent mechanical compression tests, finite element analysis (FEA) simulating postoperative and osseointegration phases, and *in vivo* validation in Beagles. The optimized BFP-C was selected for PJI model construction via hemi-hip arthroplasty (HHA), with intraoperative inoculation of *S. aureus* ranging from 250 to 10^8 colony-forming units (CFU). Longitudinal evaluation included radiography (X-ray/CT), mechanical pull-out tests, histopathology (H&E/Masson/Giemsa staining), bacterial cultures, and mobility assessments using open-field behavioural tracking.

**Results:**

BFP-C exhibited superior biomechanical compatibility, with 12.3-fold higher yield strength (6836 ± 157 N vs. 553 ± 49 N) and 97 % reduction in bone strain (0.71 % vs. 20.32 %) compared to BFP-H. All inoculated groups developed PJI with dose-dependent severity: ultra-high-dose (10^8 CFU) groups displayed severe osteolysis (pull-out strength: 24 ± 8 N vs. 924 ± 45 N in controls), biofilm formation, and mobility impairment (74 % reduction in distance travelled, 2003 ± 276 cm vs. 7976 ± 333 cm in controls), whereas low-dose (250 CFU) groups established PJI with milder manifestations, evidenced by sinus tract formation, 55.1 % reduction in pull-out strength (406 ± 15 N vs. 924 ± 45 N in controls), and concordant radiological/histopathological signs of infection. Imaging examinations revealed differential osteolytic patterns corresponding to bacterial loads. Combined wound evaluation and microbiological analyses confirmed consistent infection establishment across all replicates.

**Conclusion:**

This Beagle PJI model successfully recapitulates clinical infection dynamics, emphasizing the critical role of species-specific prosthesis design and standardized bacterial quantification. The integrated multimodal evaluation system (imaging, biomechanical, and behavioural analyses) demonstrated both the reliability of the model and its sensitivity in detecting infection progression. Its modular design supports customization for studying biofilm-resistant implants or antibiotic delivery systems. These findings not only provide a critical tool for mechanistic PJI research but also establish a theoretical foundation for clinical translation, with the quantitative multimodal framework directly informing diagnostic and therapeutic strategies.

**Translational potential:**

Beyond serving as a preclinical platform for anti-infective therapies, the model provides actionable insights into optimizing human prosthetic biomechanics, such as reducing stress shielding through FEA-informed design principles. The 3D printing workflow further demonstrates rapid prototyping capabilities for patient-specific orthopaedic implants.

## Graphical abstract

**Figure:**
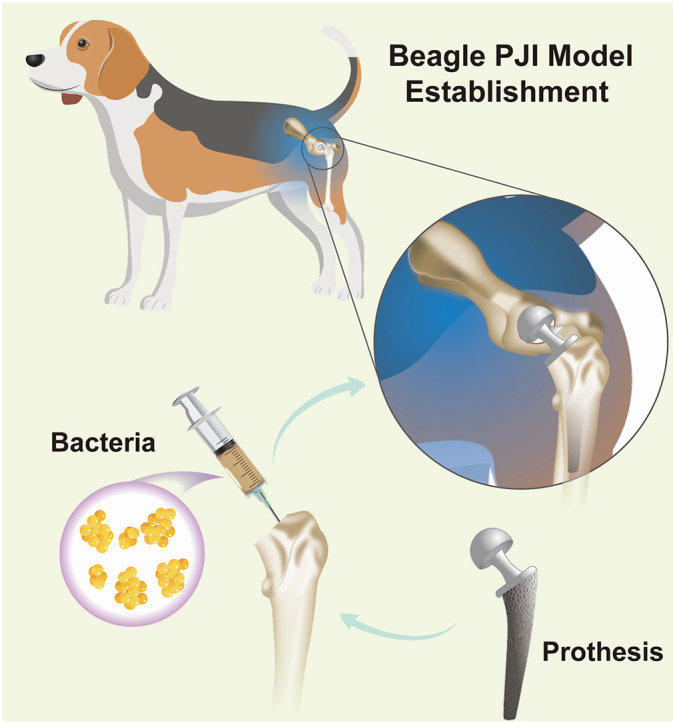

## 1 Introduction

Periprosthetic joint infection (PJI) is one of the most challenging complications following joint arthroplasty, affecting prosthetic joints in the hip, knee, elbow, and ankle [1]. Within the first two years after primary joint replacement, infection rates are approximately 9 % for elbows, about 2 % for knees, and about 1 % for hips and shoulders [2]. With an aging population, total hip and knee arthroplasty procedures are projected to increase by 659 % and 469 %, respectively, by 2060, significantly elevating the demand for revision surgeries and increasing the healthcare burden. In the United States alone, PJI-related burden is expected to reach $1.85 billion by 2030 [3,4]. Despite advancements in surgical techniques and infection control strategies, PJI treatment remains complex, often requiring multiple interventions and prolonged antibiotic therapy [5]. Therefore, establishing a reliable animal model that closely mimics clinical PJI is crucial for understanding its pathogenesis and evaluating potential therapeutic strategies [6].

Given the complexity of PJI and its clinical impact, large-animal models offer a more clinically relevant platform than small-animal models for studying the disease and testing therapeutic strategies [[7], [8], [9]]. Compared to rodents, canines have a skeletal structure, joint biomechanics, and immune response that more closely resemble those of humans, making them an ideal choice for translational research [10]. However, performing joint replacement surgery in Beagles is technically challenging due to the lack of a femoral prosthesis that fits the hip, a key limiting factor in developing a reliable Beagle PJI model.

3D printing technology, also known as additive manufacturing, creates three-dimensional objects by adding material layer by layer [11]. This method offers significant advantages in prosthesis manufacturing, enabling the rapid and precise production of complex structures [12,13]. Leveraging 3D printing allows for the precise fabrication of complex metal prostheses, offering an effective simulation tool for Beagle hip replacements that better replicate human PJI environments.

While advancements in prosthesis design through 3D printing have improved model development, another challenge in establishing Beagle PJI models is the lack of standardized bacterial loading methods and reference standards for bacterial quantities. In current implant-related infection models, bacterial loads range from 10^2 to 10^9 colony-forming units (CFU), varying even within the same animal species [14]. Although Petty et al. explored the impact of different bacterial quantities and implant materials on infection rates in dogs in 1985 [15], no clear standard for bacterial loads in canines has since been established.

**Scheme 1:**
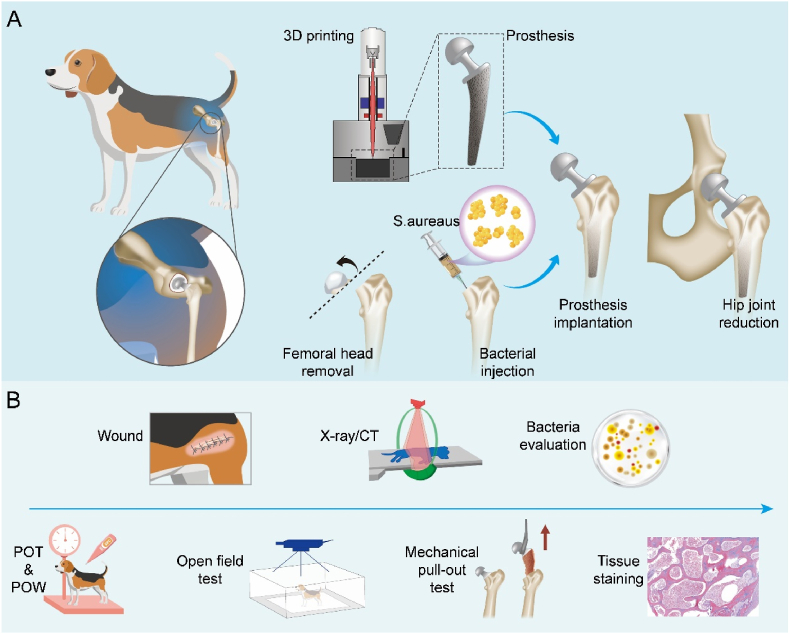
(A) Design, 3D printing of prosthesis and construction of the Beagle model of hip PJI. A femoral head prosthesis was designed and 3D-printed to match the Beagle hip anatomy. Subsequently, Beagles underwent HHA with *S. aureus* injected into the medullary cavity prior to femoral prosthesis implantation. (B) Postoperative infection assessment. Infection was assessed by monitoring postoperative body temperature (POT) and body weight (POW), evaluating wound healing, performing the open field test, conducting X-ray and CT imaging, and analyzing bacterial load, mechanical pull-out strength, and histological staining to determine the severity of hip PJI.

To address these challenges, we designed two femoral prostheses suitable for Beagle hip anatomy. Through mechanical testing, finite element analysis, and *in vivo* fixation validation, we selected the optimal prosthesis for constructing the Beagle PJI model. The model was established by performing hemi-hip arthroplasty (HHA) and injecting varying concentrations of *Staphylococcus aureus* (*S. aureus*, 250 to 10^8 CFU) into the femoral canal before implant insertion. Postoperative infection severity was evaluated through a multimodal assessment protocol combining radiographs, histology, bacterial cultures, and mechanical pull-out tests (Scheme 1). Results showed that all bacterial concentrations with the prioritized BFP induced PJI, providing valuable insights for future Beagle PJI research and supporting the *in vivo* validation of antibacterial materials, thereby aiding clinical translation.

## 2 Materials and methods

### 2.1 Animals and experimental design

This study was reviewed and approved by the Animal Experimental Ethics Committee (Ref. No. YSY-DWLL-2023516). Beagle dogs aged 12 months on average (ranging from 10 to 14 months) and weighing approximately 13 kg (ranging from 12 to 15 kg) were used. The experiment consisted of two phases: the prosthesis evaluation and the PJI model construction. In the first phase, two types of Beagle femoral prostheses (BFP), Canine-optimized (BFP-C) and Human-derived (BFP-H), were implanted into 6 cadaveric and 6 live Beagle dogs (BFP-C vs. BFP-H, n = 3 per group) for comparative analysis. Based on biomechanical performance and anatomical compatibility, the optimal design was selected for the subsequent PJI model construction. In the second phase, 15 animals were divided into five groups (n = 3 per group) based on bacterial inoculation: a control group receiving PBS only (CON), and four experimental groups challenged with increasing doses of *S. aureus*—250 CFU (low-dose bacterial group, LD), 1000 CFU (middle-dose bacterial group, MD), 10^6 CFU (high-dose bacterial group, HD), and 10^8 CFU (ultra-high dose bacterial group, UHD).

### 2.2 Design and manufacture of BFP and surgical instruments

Preoperative CT scans were performed on Beagle dog hips to obtain detailed anatomical data. The obtained imaging data were imported into Mimics software for femoral reconstruction. Based on these reconstructed femur models, two types of BFP were designed. The first design, BFP-C, was tailored to the anatomical and medullary structures of the Beagle dog, and BFP-H was a scaled-down version of a commercially used human femoral prosthesis. Both designs were based on critical dimensions, including femoral head diameter (D), cervical-diaphyseal angle (CDA), neck length (L), stem height (H), and neck width (W), to ensure anatomical accuracy. The final prosthesis models were fabricated using titanium alloy (Ti-6Al-4V) via electron beam melting (EBM) 3D printing technology. Additionally, specialized surgical instruments, including medullary reamers and a prosthesis angle indicator, among others, were designed and manufactured to support the Beagle HHA procedure.

### 2.3 Mechanical performance evaluation and finite element analysis of BFP-C and BFP-H prostheses

The mechanical properties of BFP-C and BFP-H were evaluated using an Instron universal testing machine. Axial compression tests were conducted at a displacement rate of 2 mm/min, applying a uniaxial load to the distal stem of each prosthesis until deformation occurred. Key parameters, including structural stiffness and yield strength, were recorded to compare the mechanical performance of the two designs.

A three-dimensional finite element model (FEM) was developed to evaluate the biomechanical behavior of the implanted prostheses in both early post-implantation and long-term osseointegration phases. A 3D femoral model was reconstructed from Beagle CT scan data (DICOM format) and imported into Geomagic Studio 2017 for geometric smoothing and optimization. The refined model was then assembled with the prostheses (BFP-C and BFP-H) to generate the femur-prosthesis system. Meshing was performed using tetrahedral elements in Hypermesh 13.0 (Altair, USA), and the resulting models were exported in INP format for analysis in ABAQUS (Dassault Systèmes, Franch). Material properties were assigned based on Table 1, modeling the prostheses as Ti-6Al-4V titanium alloy and segmenting the femur into cortical and trabecular bone based on grayscale values. Both the prosthesis and femur were assumed to be isotropic and linear elastic. For FEA simulations, two conditions were analyzed: early post-implantation and long-term osseointegration. In the early phase, the femur-prosthesis interface was defined as a frictional contact with a coefficient of 0.3, while in the late phase, the interface was assumed to be fully bonded. The distal femur was fully constrained, with a 200 N axial load applied to the femoral head of the prosthesis to simulate physiological loading conditions.

**Table 1:** Material parameters used for the FEA simulation in this study.

Material	Young's modulus (GPa)	Poisson's ratio (dimensionless)
Beagle bone (cortical)	13	0.3
Beagle bone (cancellous)	1	0.3
Prosthesis (Ti-6Al-4V)	110	0.3

### 2.4 Beagle HHA with BFP-C and BFP-H

**Fig. 1:**
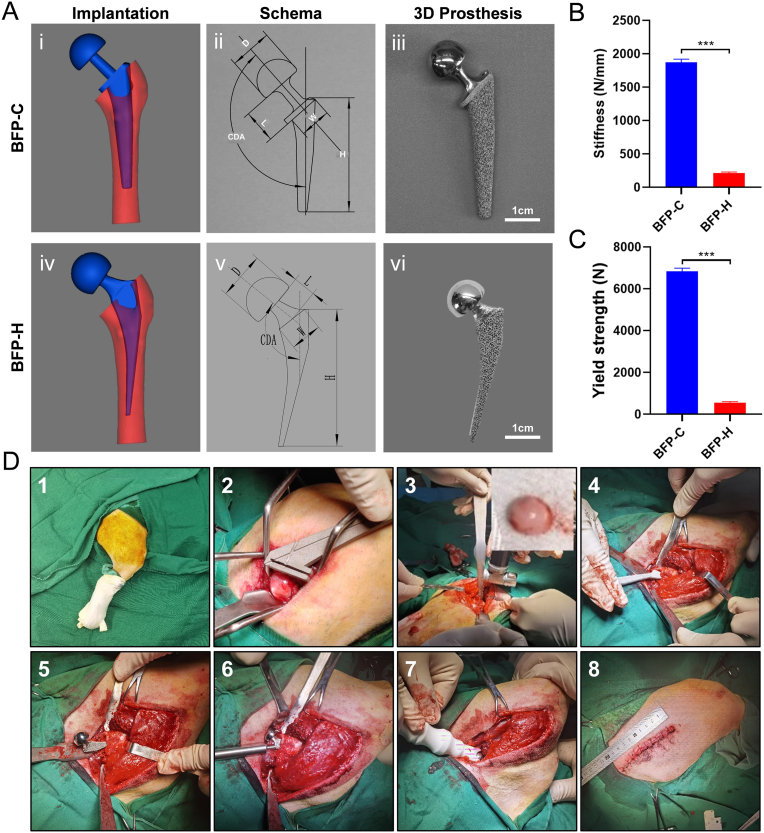
Design, 3D printing, and implantation of BHP-C & BFP-H. (A) Design and 3D printing of BFP-C and BFP-H. (i-vi) i and iv, Femoral modeling and prosthesis fitting simulation; ii and v, BFP schema showing key parameters: D (femoral head diameter), L (neck length), H (stem height), W (neck width), and CDA (cervical-diaphyseal angle); iii and vi, 3D-printed custom-designed BFP prosthesis. (B) BFP stiffness. (C) BFP yield strength. (D) Hemi-hip arthroplasty procedure (HHA): (1) Disinfect and drape the surgical site; (2) Expose the femoral head; (3) Resect the femoral head; (4) Broach the femoral canal; (5) Implant BFP-C or BFP-H prosthesis; (6) Secure the prosthesis and confirm alignment; (7) Reduce the hip joint; (8) Close the surgical site. Data are presented as mean ± SD (∗*P* < 0.05, ∗∗*P* < 0.01, ∗∗∗*P* < 0.001; n=3).

Anesthesia was induced in Beagle dogs using ketamine (10 mg/kg) and xylazine (0.5 mg/kg), followed by endotracheal intubation. Isoflurane was then used to maintain anesthesia. The hip joint area was shaved and disinfected with povidone-iodine solution. A 10–15 cm incision was made along the right femoral axis, and the muscles (gluteus maximus, gluteus medius, piriformis, biceps femoris, and teres minor) were dissected to expose the hip joint. The femoral head was removed with an electric saw, and the marrow cavity was reamed. The BFP-C or BFP-H was implanted under the guidance of BFP angle indicator, and the hip joint was reduced. The incision was closed in layers (Fig. 1D). Postoperative analgesia was provided to manage pain, while antibiotics were not administered, and Elizabethan collars were used to prevent licking or scratching the wound.

### 2.5 Mechanical fixation strength and bone ingrowth analysis of prostheses after implantation

X-ray imaging was performed on postoperative day (POD) 0 to evaluate the prosthesis position and alignment with the hip joint. X-ray and CT scans were repeated on POD 28 to assess long-term fixation and bone ingrowth. To assess fixation strength and bone ingrowth, pull-out tests were performed, which was performed on an Instron universal testing machine. Briefly, the femur was fixed distally with bone cement, and the prosthesis was held with a custom fixture. The pull-out test was performed vertically at a speed of 2 mm/min, and the maximum pull-out force (in Newtons, N) was recorded. For short-term fixation, the test was conducted immediately after implanting the prosthesis in the Beagle cadavers. For long-term fixation, the test was performed on femoral specimens from euthanized Beagles on POD 28.

### 2.6 Comparison of in vitro experiments between gram-negative bacteria - *Escherichia coli* (*E*. *coli*) and gram-positive bacteria -*S. aureus*

In order to test the adhesion ability and activity of different bacterial species and concentrations on the prosthesis, a porous titanium alloy scaffold (the same as the prosthesis material) and different bacterial concentrations (0, 250, 1000, 10^6 and 10^8 CFU/3 mL medium) were co-cultured with *S. aureus* or *E*. *coli* at 37 °C for 6 h. The scaffolds were observed under a scanning electron microscope (SEM), and the bacteria were detached from the scaffolds for plate coating. To verify the effect of different bacterial species and concentrations on the state and activity of the cells, MSCs or GFP-tagged inducible immune and matrix regulatory cells (IMRCs) were seeded into 24-well plates and grown to 70–80 % confluence. Then, *E. coli* or *S. aureus* suspension was added to the well (0, 250, 1000, 10^6, and 10^8 CFU were added to 100 μl culture medium, respectively). After 6 h of co-culture, the cell morphology was observed under a fluorescence microscope.

### 2.7 Preparation of the bacteria and establishment of the Beagle PJI model

Based on previous mechanical and fixation evaluations, BFP-C was selected for subsequent Beagle PJI model establishment. *S. aureus* subsp. aureus ATCC®29213 was cultured in tryptic soy broth at 37 °C with 5 % CO_2_ under constant shaking at 200 rpm and harvested at the mid-log phase (OD600 ≈ 0.6). The culture was centrifuged at 4000×*g* for 10 min, and the pellet was washed twice with sterile PBS. The bacterial concentration was adjusted to 1.5 × 10^9 CFU/ml using a spectrophotometer and verified by serial dilution plating. On the day of infection, the bacterial suspension was diluted in sterile PBS to obtain the required concentrations (200 μl aliquots containing 250, 1000, 10^6, and 10^8 CFU) for *in vivo* infection.

The HHA procedure was performed as described in Section 2.4.

### 2.8 Assessment of body temperature and weight

Body temperature and weight were recorded at multiple time points preoperatively and postoperatively to evaluate changes over time. Rectal temperature was recorded preoperatively and at infection POD 1, 3, 7, 14, 21, and 28. Body weight was monitored at POD 3, 7, 14, 21, and 28 to evaluate weight fluctuations after surgery.

### 2.9 Evaluation of mobility using the open field test (OFT)

Beagle mobility was assessed in a designated testing arena (150 cm × 260 cm) at POD 28. Each Beagle was placed in the center of the arena and allowed to explore freely for 10 min. A camera was positioned above the arena to record Beagle activity. Video recordings from 3 to 8 min of each test were analyzed quantitatively using EthoVision XT (Noldus Information Technology, The Netherlands). The arena was cleaned and dried before and after each test to ensure consistency.

### 2.10 Radiographic assessment

Radiographic assessments, including X-ray and CT, were performed at POD 0, 14, and 28 by three independent experts to evaluate bone quality, periosteal reaction, implant positioning, and soft tissue swelling or fluid accumulation. Radiographic scoring was conducted based on these parameters (Table S1).

### 2.11 Wound evaluation

Wound appearance and sinus tract discharge were first observed. After euthanasia and shaving the wound area, the wound healing was further assessed. The original surgical incision was reopened following disinfection to evaluate subcutaneous exudation. Wound healing in different Beagle groups was evaluated by three experts using a wound scoring system (Table S2). Sterile swabs were then used to collect exudate for bacterial identification to detect potential cross-infections.

### 2.12 Bacterial identification of peri-hip joint tissues and BFP surface

After reopening the wound, muscle tissue was separated to expose the hip joint, and five tissue samples (∼100 mg each) were collected. The BFP were removed from the femur under sterile conditions and placed into a 50 ml centrifuge tube with 30 ml saline. Both tissue samples and BFP were washed in saline, then homogenized in 900 μl saline (for tissues) or shaken thoroughly (for BFP) to release bacteria. The homogenates were filtered through a sterile mesh to remove large debris. The filtered liquids were diluted 50-fold (for tissues) or 10^5-fold (for BFP), and 50 μl of each dilution was spread on solid culture media using glass beads. The plates were sealed and incubated at 37 °C for 24–48 h. Colony images and counts were recorded using a colony counter to evaluate colony numbers and morphology.

### 2.13 Histological analysis of infected bone tissue

Fixed bone tissues were decalcified in 20 % EDTA for 2 months. The tissues were then dehydrated in a graded ethanol series, cleared with xylene. After processing, the samples were embedded in paraffin, sectioned, and stained with hematoxylin-eosin (H&E), Masson's trichrome, and Giemsa staining to evaluate infection severity and tissue response. Quantitative analysis was performed using ImageJ software (National Institutes of Health, USA). H&E staining was used to quantify the number of inflammatory cells per high-power field (HPF). Masson's trichrome staining was assessed using a scoring system based on collagen deposition, new bone formation, bone matrix damage, and fibrosis degree (Table S3). Giemsa staining was used to quantify the number of *S. aureus* per HPF.

### 2.14 Statistics

Statistical analyses were conducted using GraphPad Prism 8 software. Comparisons between experimental and CON groups were performed using one-way ANOVA followed by Dunnett's multiple comparisons test, while comparisons among all groups were conducted using one-way ANOVA followed by Tukey's multiple comparisons test. For comparisons between two groups, an unpaired t-test was used. Results are presented as mean ± standard deviation (SD). Statistical significance was defined as *P* < 0.05. The representation of statistical significance was denoted as follows: ∗*P* < 0.05, ∗∗*P* < 0.01, ∗∗∗*P* < 0.001.

## 3 Results

### 3.1 Successful construction of BFP-C & BFP-H prostheses and HHA surgical tools

CT scans of the Beagle hip joints (Fig. S1) were used to design two custom prostheses (Fig. 1A), with key parameters summarized in Table 2. These prostheses were fabricated using EBM technology. Mechanical strength testing was performed (Fig. S2), showing that BFP-C exhibited 8.7-fold higher structural stiffness (1872 ± 48 N/mm vs. 215 ± 15 N/mm, *P* < 0.001, unpaired t-test) and 12.3-fold greater yield strength (6836 ± 157 N vs. 553 ± 49 N, *P* < 0.001) compared to BFP-H (Fig. 1B–C). Subsequently, we verified the adhesion ability and activity of Gram-negative (e.g., *E*. *coli*) and Gram-positive (e.g., *S. aureus*) bacteria on the prosthesis. The results showed that no clear strain specific preference on the prosthetic scaffold was observed, however, as bacterial concentrations consistently increased (Fig. S3), quantitative analysis of bacteria shedding from the scaffold showed that the bacterial load on the implant also increased with inoculum size (Fig. S4), indicating that adherent bacteria maintained their proliferative capacity. Bacterial adhesion was mainly determined by dose-dependent relationship rather than bacterial type. Furthermore, the effects of the two bacteria on the state and activity of the cells were verified. The results showed that increasing bacterial concentration led to progressive deterioration of cell morphology, cell rounding and loss of adhesion to medium quality (Fig. S5) and a significant increase in the proportion of dead cells (Fig. S6), indicating that the effects of both bacteria on cell morphology and activity were similar. Therefore, *S. aureus*, which is more common in clinical infections, was used for subsequent studies in animals. Additionally, specialized surgical tools for Beagle HHA were also designed and fabricated (Fig. S7). Subsequently, these tools were used to conduct HHA on Beagle (Fig. 1D).

**Table 2:** Key parameters of the two prostheses.

Parameter	D (mm)	CDA (°)	L (mm)	W (mm)	H (mm)
BFP-C	15.8	138°	17.7	13.4	50
BFP-H	15.8	138°	9.0	12.8	50

Note: D (femoral head diameter), L (neck length), H (stem height), W (neck width), and CDA (cervical-diaphyseal angle).

### 3.2 FEA comparison of biomechanical properties of the two prostheses

**Fig. 2:**
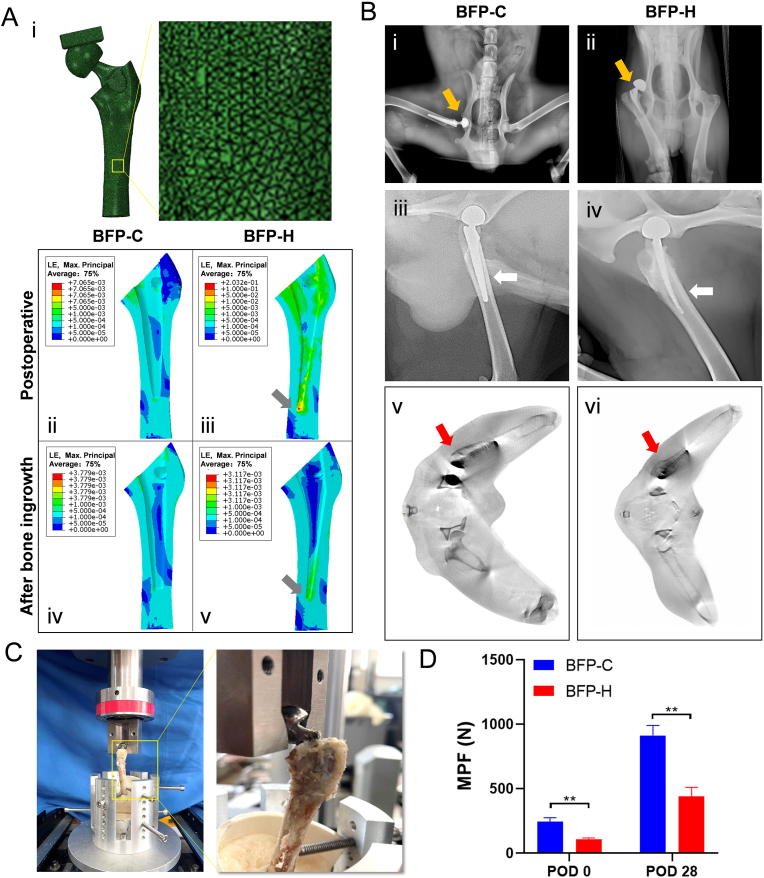
Performance comparison of two prostheses *ex vivo* & *in vivo*. (A) Finite element analysis of BFP-C and BFP-H. (i–v) i, Mesh generation; ii-iii, short-term deformation for BFP-C and BFP-H; iv-v, long-term deformation for BFP-C and BFP-H. (B) Postoperative imaging data. (i-vi) i, BFP-C at POD 0, with proper prosthesis positioning; ii, BFP-H at POD 0, femoral head dislocation (yellow arrow); iii, BFP-C at POD 28, with proper prosthesis positioning; iv, BFP-H at POD 28, showing BFP-H distal deformation (white arrow); v-vi, CT imaging of BFP-C and BFP-H at POD 28, prostheses within the femoral canal. (C) Biomechanical pull-out test. Pull-out test images, with distant and close-up views. (D) Maximum pull-out forces at POD 0 and POD 28. Data are presented as mean ± SD (∗*P* < 0.05, ∗∗*P* < 0.01, ∗∗∗*P* < 0.001; n = 3).

The femoral-prosthesis system was divided into mesh elements using adaptive meshing (Fig. 2Ai) to capture stress and strain distributions in critical areas while reducing computational load in non-critical regions. Simulations of both short-term (postoperative) and long-term (bone ingrowth) mechanical responses were conducted for the prostheses (Fig. 2Aii-v). The simulations revealed that the maximum strain around the bone in the BFP-C group was 0.71 % postoperatively, while the maximum strain in the BFP-H group was 20.32 %. Post-bone ingrowth, the peak strain for BFP-C decreased to 0.38 %, while for BFP-H it was 0.31 %. Initially, BFP-H exhibited higher bone strain, suggesting potential damage to the surrounding bone, while both BFP-C and BFP-H showed reduced and comparable bone strains after long-term implantation. Notably, the BFP-H tip displayed a distinct high-strain area in both postoperative and bone ingrowth simulations (indicated by the gray arrows), which could suggest higher bone damage. These results suggest that BFP-C, with optimized stiffness and interface design, offers superior biomechanical adaptation for both early and long-term stability.

### 3.3 Comparison of BFP performance post-implantation

To assess implantation effects and validate FEA, BFP-C and BFP-H were implanted in Beagles. All animals successfully underwent right-sided HHA. X-rays on POD 0 confirmed accurate BFP-C implantation into the femoral medullary cavity, with no hip dislocation observed (Fig. 2Bi). In contrast, one BFP-H prosthesis showed femoral head dislocation (Fig. 2Bii). At POD 28, bone ingrowth was observed in the BFP-C group, and the prosthesis remained secure (Fig. 2Biii). In the BFP-H group, distal deformation of the femoral stem was observed (Fig. 2Biv), matching the FEA result of BFP-H distal deformation (Fig. 2Av). At POD 28, the CT imaging results revealed that the prostheses within the femoral canal in the BFP-C group of Beagle dogs were well-positioned, with femoral stem centered and showing no significant deviation. In contrast, the prostheses in the BFP-H group of Beagle dogs exhibited noticeable misalignment (Fig. 2Bv-vi).

Furthermore, biomechanical pull-out test was conducted on the two types of implantations respectively (Fig. 2C). BFP-C demonstrated 2.2-fold higher pull-out strength than BFP-H at POD 0 (245 ± 31 N vs. 109 ± 10 N, *P* < 0.001) and 2.1-fold higher at POD 28 (913 ± 78 N vs. 440 ± 70 N, *P* < 0.001) (Fig. 2D). Based on these findings, BFP-C was selected for subsequent PJI model construction.

### 3.4 Postoperative conditions and mobility assessment

**Fig. 3:**
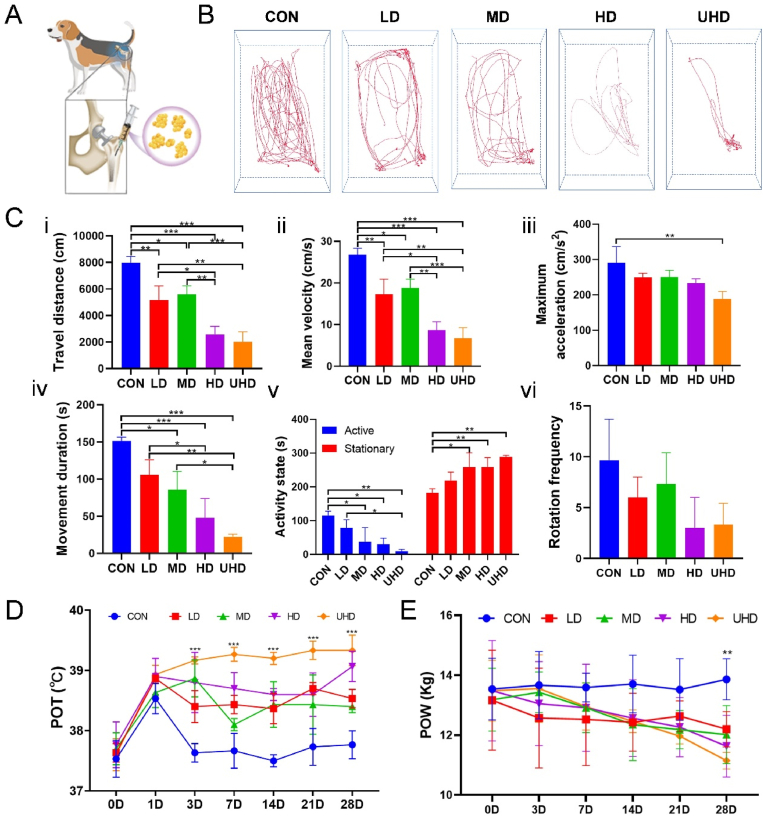
Beagle PJI model: postoperative temperature, body weight, and open field test results. (A) Schematic of the Beagle PJI model establishment. (B) Representative trajectory images from the open field test. (C) Quantitative analysis of trajectory data, including (i) distance travelled, (ii) average speed, (iii) maximum acceleration, (iv) duration of movement, (v) activity level, and (vi) rotation frequency. (D) POT and (E) POW in different days. Data are presented as mean ± SD (∗*P* < 0.05, ∗∗*P* < 0.01, ∗∗∗*P* < 0.001; n = 3).

The Beagle PJI models were established by injecting different dose of *S. aureus* into the surgical site before prosthesis implantation (Fig. 3A). An OFT was conducted to analyze the locomotion change at POD 28. The CON group showed excellent mobility recovery (Video S1). OFT result reveals a dose-dependent reduction of locomotor ability after bacterial infection (Fig. 3B–C). Specifically, the CON group had the highest values in all mobility indicators, with travel distance of 7976 ± 333 cm and mean velocity of 26.8 ± 1.6 cm/s (Fig. 3Ci-ii). The LD/MD groups showed decreased mobility, with travel distances of 5155 ± 1073 cm and 5602 ± 629 cm and mean velocities of 17.3 ± 3.6 cm/s and 18.8 ± 2.1 cm/s, while HD/UHD groups exhibited more significant declines, with in travel distance of 2581 ± 598 cm and 2003 ± 276 cm and mean velocities of 8.7 ± 2.0 cm/s and 6.7 ± 2.5 cm/s (Fig. 3Ci-ii). Similarly, maximum acceleration decreased from 290.6 ± 45.8 cm/s2 in the CON group to 188.5 ± 21.7 cm/s^2^ in the UHD group (Fig. 3Ciii), and movement duration declined from 151.8 ± 5.0 s to 22.6 ± 3.5 s. Although no significant difference in rotation frequency was noted, HD/UHD groups had lower average frequencies (3.0 ± 3.0 in HD group and 3.3 ± 2.1 in UHD group) (Fig. 3Cvi). The CON group also exhibited higher activity levels (116.0 ± 12.4 s in CON group vs. 9.5 ± 5.6 s in UHD group), while the experimental groups remained more stationary (181.7 ± 12.7 s in CON group vs. 288.2 ± 5.6 s in UHD group), with activity inversely correlated with bacterial inoculation (Fig. 3Cv).

POT rose on POD 1, with experimental groups showing sustained fever, which was dose-dependent, particularly in the UHD group. The CON group returned to normal temperature by POD 3 (Fig. 3D). POW changes indicated weight loss in all experimental groups, correlated with bacterial dose, with the UHD group showing the most significant loss. In contrast, the CON group gained weight slightly (Fig. 3E).

### 3.5 Radiographic and mechanical pull-out evidence of PJI

**Fig. 4:**
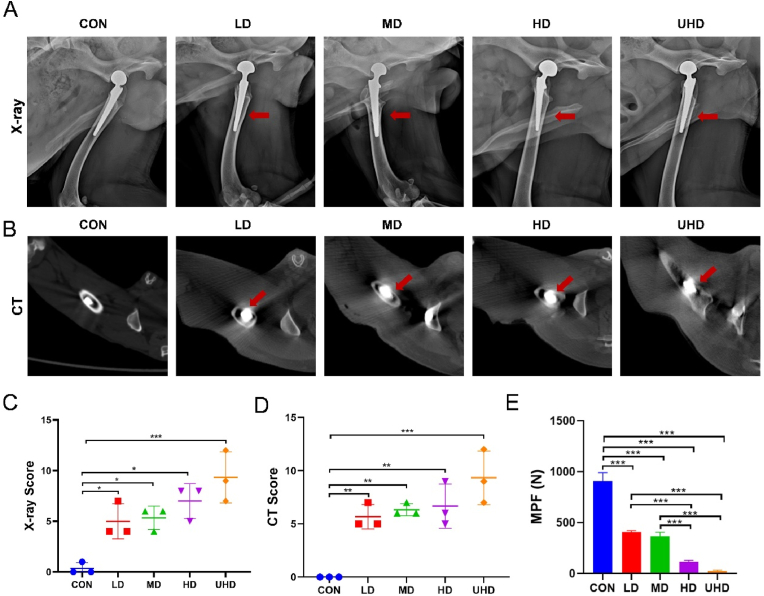
Postoperative imaging evaluation and maximum pull-out test. (A) X-ray images of PJI model groups on POD 28, with red arrows indicating changes in medullary bone structure. (B) CT images of PJI model groups on POD 28, with red arrows indicating bone destruction. (C) X-ray image scoring on POD 28. (D) CT image scoring on POD 28. (E) Maximum pull-out forces on POD 28 after infection. Data are presented as mean ± SD (∗*P* < 0.05, ∗∗*P* < 0.01, ∗∗∗*P* < 0.001; n = 3).

Radiological findings from X-ray and CT scans were consistent, though X-ray images showed infection signs later than CT scans. By POD 28, both X-ray and CT images revealed infection markers. Bone destruction, periosteal reactions (indicated by red arrows), and soft tissue swelling or fluid accumulation around the BFP-C were observed from the LD to the UHD groups (Fig. 4A–B). In contrast, the CON group showed bone ingrowth and well-integrated prosthesis–bone interfaces on both X-ray and CT images. X-ray and CT image scores on POD 28 were evaluated, and the scores in the LD group were significantly lower than those in the UHD group (Fig. 4C–D). Additionally, mechanical pull-out strength at POD 28 was 97.4 % lower in the UHD group (24 ± 8 N) and 86.8 % lower in the HD group (120 ± 11 N) compared to controls (924 ± 45 N, p < 0.001 for both, one-way ANOVA with Tukey's test). The LD and MD groups exhibited moderate reductions (55.1 % [410 ± 15 N] and 59.5 % [370 ± 28 N], respectively), but their difference was not statistically significant (*P* = 0.37) (Fig. 4E).

### 3.6 Wound and bacterial infection assessment

**Fig. 5:**
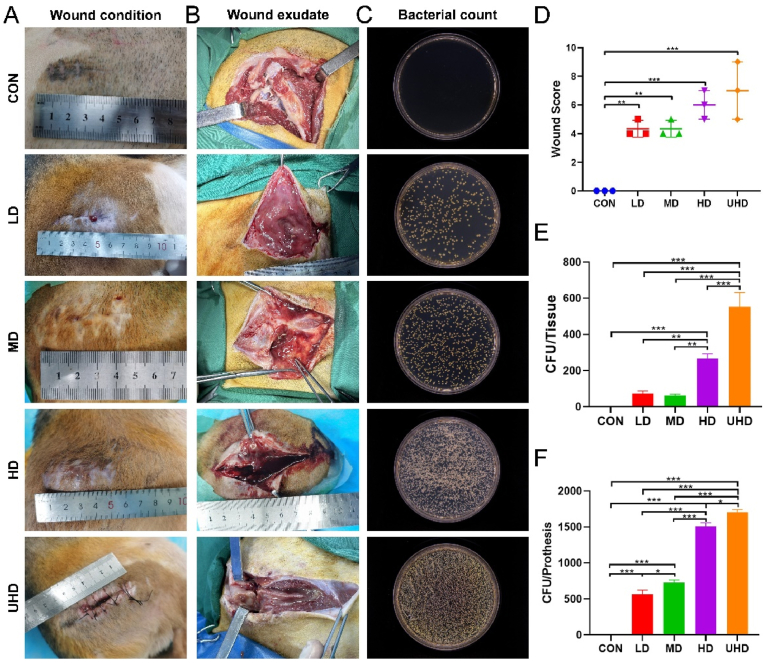
Wound evaluation and bacterial detection. (A) Appearance of the surgical wound on POD 28. (B) Subcutaneous exudate of the wound. (C) Representative CFU counts of *S. aureus* on the BFP-C surface. (D) Wound scoring. (E) Semi-quantification of CFU counts on the BFP-C surface (n = 3). (F) Semi-quantification of CFU counts in periarticular tissue (n = 5). Data are presented as mean ± SD (∗*P* < 0.05, ∗∗*P* < 0.01, ∗∗∗*P* < 0.001).

The CON group exhibited dry, healing wounds without signs of infection. In contrast, all experimental groups, including the LD group, showed redness, swelling, sinus tracts, and significant infection, with the UHD group presenting large unhealed areas (Fig. 5A). In the postoperative wound assessment, the UHD group achieved the highest score, while the LD group received the lowest score among the experimental groups (Fig. 5D). Upon opening the wounds, the CON group showed healthy subcutaneous blood supply and no infection, while exudate was present in the experimental groups, with the volume correlating to the bacterial inoculation dose (Fig. 5B). Semi-quantitative wound scoring revealed significant infection in all experimental groups compared to the CON group (Fig. 5C). Notably, one Beagle in the LD group developed a sinus tract and purulent discharge (Video S2). Bacterial cultures from the BFP-C surfaces were positive in all experimental groups, with bacterial counts correlating with the inoculation dose. Significant differences were observed between the HD/UHD groups and the LD/MD groups (Fig. 5E). Bacterial cultures from the hip joint tissues mirrored the findings from the femoral head prostheses, with HD/UHD groups showing more severe infection (Fig. 5F). Exudate analysis identified *S. aureus* as the infecting bacterium, with no other pathogens detected (Fig. S8).

### 3.7 Histological analysis of bone destruction and infection

**Fig. 6:**
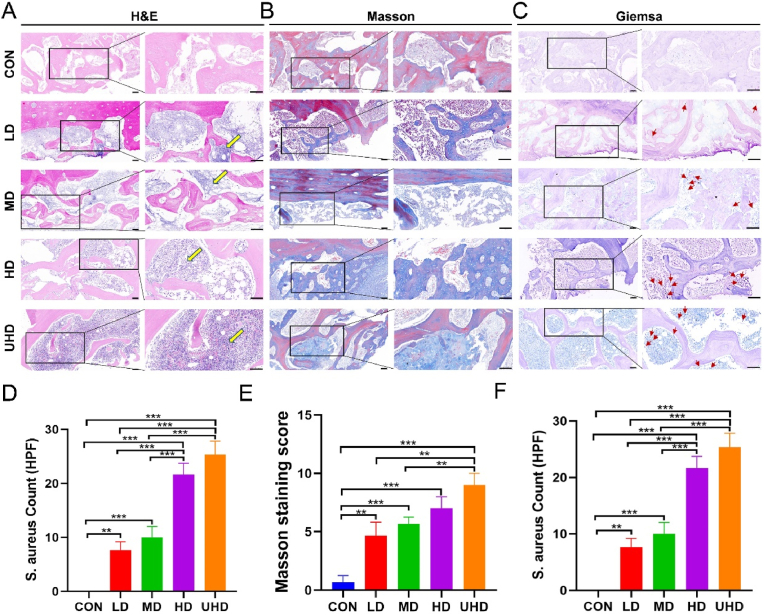
Histological evaluation of bone infection. (A) H&E staining of Beagle femurs, yellow arrows indicate inflammatory cells. (B) Masson's trichrome staining of Beagle femurs. (C) Giemsa staining of Beagle femurs, red arrows indicate *S. aureus* and biofilms. (D) Inflammatory cell count per high-power field. (E) Masson staining scores. (F) *S. aureus* count per high-power field. Scale bar, 100 μm. Data are presented as mean ± SD (∗*P* < 0.05, ∗∗*P* < 0.01, ∗∗∗*P* < 0.001; n = 6).

H&E staining showed intact bone structure in the CON group, with no inflammation. In contrast, experimental groups displayed bone destruction and inflammatory infiltration, most notably in the UHD group (Fig. 6A). The UHD group exhibited the highest number of inflammatory cells counted under high-power field microscopy (Fig. 6D). Masson staining revealed normal bone structure in the CON group, while experimental groups exhibited poor collagen deposition, disrupted bone matrix, and reduced new bone formation (Fig. 6B). Masson scores, based on collagen, new bone, matrix damage, and fibrosis, showed significant differences between experimental and CON groups, correlating with bacterial load (Fig. 6E). Giemsa staining revealed abundant bacteria and biofilms in the HD/UHD groups, moderate growth in the LD/MD groups, and no bacteria in the CON group (Fig. 6C). The number of *S. aureus* counted per high-power field was significantly higher in the HD/UHD groups compared to the LD/MD groups (Fig. 6F).

## 4 Discussion

PJI remains a principal cause of arthroplasty failure, underscoring the need for preclinical models that recapitulate clinical infection dynamics. This study established a standardised Beagle PJI model integrating species-specific 3D-printed prostheses (BFP-C) with graded *S. aureus* inoculation (250–10^8 CFU). Our findings highlight three critical advances: (1) the biomechanical superiority of anatomically optimized prostheses, (2) a nonlinear dose-dependent relationship between bacterial load and infection severity, and (3) the diagnostic and prognostic value of a multimodal evaluation system. These innovations collectively bridge translational gaps in PJI research while offering actionable insights for clinical practice.

The BFP-C prosthesis, designed using CT-derived canine femoral parameters (D: 15.8 mm; CDA: 138°, L: 17.7 mm, W: 13.4 mm, H: 50 mm), demonstrated exceptional biomechanical compatibility. Its yield strength (6836 ± 157 N) and postoperative stability (pull-out strength: 913 ± 78 N) far exceeded those of the human-scaled BFP-H (553 ± 49 N; 440 ± 70 N, *P* < 0.001). Finite element analysis (FEA) revealed that BFP-C reduced peri-implant bone strain to 0.71 % during osseointegration—28-fold lower than BFP-H (20.32 %)—thereby minimizing stress shielding and microdamage. By POD 28, bone strain further decreased to 0.38 %, aligning with clinical osseointegration benchmarks [16]. In contrast, BFP-H induced progressive bone resorption due to distal high-strain zones, reducing pull-out strength by 55 % (*P* < 0.001). Notably, one BFP-H-implanted Beagle experienced prosthesis dislocation, emphasizing the risks of using human-derived designs in animal models. These results validate species-specific prostheses as essential for replicating human arthroplasty biomechanics while maintaining implant stability—a prerequisite for reliable PJI modelling.

Given that *S. aureus* accounts for 22.6–37.9 % of clinical PJI cases and represents the most common pathogen among Gram-positive bacteria, its use in this study aligns with epidemiological data and established preclinical models [[17], [18], [19], [20]]. In contrast, Gram-negative organisms such as *E. coli* are responsible for a significantly smaller proportion of PJIs (2.3–7.89 %) [21,22] and pose challenges in large-animal models due to their propensity to trigger severe endotoxin-mediated systemic responses. These factors reduce model reproducibility and raise ethical concerns. Furthermore, in vitro assays showed comparable pathogenic effects between *S. aureus* and *E. coli* in terms of implant adhesion, cell viability reduction, and cytotoxicity, supporting the selection of *S. aureus* as a clinically relevant and experimentally stable agent for model development.

The established model uniquely quantified the impact of bacterial load on PJI progression. All experimental groups (≥250 CFU) developed infections, with severity escalating nonlinearly with dose. Ultra-high-dose (UHD: 10^8 CFU) animals exhibited near-complete osseointegration failure (pull-out strength: 24 ± 8 N vs. 924 ± 45 N controls, *P* < 0.001), severe osteolysis, and biofilm formation. Histopathology revealed bacterial densities of 25 ± 2 CFU/HPF (Giemsa staining) and extensive bone matrix collapse (Masson score: 9 ± 1 vs. 0.67 ± 0.58 controls, *P* < 0.001), suggesting quorum sensing-mediated virulence factor upregulation [23]. Even low-dose (LD: 250 CFU) inoculations induced PJI, evidenced by sinus tract formation, a 55.1 % pull-out strength reduction (406 ± 15 N vs. 924 ± 45 N, *P* < 0.001), and concordant radiological/histopathological signs. This finding aligns with the seminal work of Elek et al. [24], where 100 CFU sufficed to trigger implant-associated infections, underscoring the pivotal role of foreign bodies in lowering infection thresholds. Longitudinal monitoring further identified dose-dependent systemic responses (e.g., UHD: peak temperature 39.6 °C, 17 % weight loss), offering quantifiable early-warning biomarkers for clinical PJI.

A key innovation lies in the integration of imaging, biomechanical, and behavioural analyses to overcome limitations of conventional methods. CT detected periosteal reactions in UHD animals 14 days earlier than X-ray. Mechanical pull-out tests provided objective infection metrics (e.g., 97.4 % strength loss in UHD), enabling quantitative evaluation of antimicrobial interventions. Behavioural tracking further linked functional impairment to infection severity: UHD animals travelled 74 % shorter distances (2003 ± 276 cm vs. 7976 ± 333 cm controls, *P* < 0.001), mirroring human PJI gait disturbances. This multimodal framework not only enhances diagnostic sensitivity but also supports therapeutic efficacy assessments, such as evaluating antibiotic-loaded bone cement on functional recovery.

The 3D-printed workflow demonstrated rapid prototyping capabilities for patient-specific implants—a critical asset for revision surgeries. FEA-guided strain optimization (BFP-C: 0.38 % post-osseointegration) paves the way for low-modulus gradient porous prostheses to mitigate stress shielding. Furthermore, this model serves as a robust platform for testing novel anti-biofilm strategies (e.g., vancomycin-polydopamine nano-coatings) and localized antibiotic delivery systems.

Despite successfully replicating key clinical features of PJI, this study has several limitations. The model employed only *S*. *aureus* as the infective agent, without inclusion of other clinically relevant pathogens such as Gram-negative bacteria (*Pseudomonas aeruginosa*, *E*. *coli*), and overlooked polymicrobial infections, which are prevalent in approximately 19 % of clinical PJI cases [25]. In addition, the observation period was limited to POD 28, restricting the evaluation of long-term outcomes such as chronic inflammation, delayed immune responses, and tissue regeneration. The relatively small number of animals per group may also limit statistical robustness. Future research will focus on elucidating key pathogenic mechanisms including biofilm formation, immune evasion, and osteolysis, while leveraging this model to assess the *in vivo* efficacy and safety of novel antimicrobial materials, antibiotic delivery systems, and immunomodulatory strategies to enhance translational relevance.

## 5 Conclusion

This study establishes the first Beagle PJI model combining species-specific prostheses, quantitative bacterial dosing, and multimodal evaluation. The BFP-C design highlights the necessity of anatomic compatibility in PJI modeling, while dose-dependent infection dynamics provide a framework for clinical decision-making, such as defining critical bacterial thresholds. The 3D-printed workflow and FEA-guided optimization strategies offer direct pathways for developing patient-specific implants with enhanced biomechanics. By bridging preclinical and clinical PJI research, this platform accelerates the translation of novel anti-infective therapies and diagnostic innovations, ultimately improving outcomes in joint arthroplasty.

**Table:** Abbreviations

BFP	beagle femoral prosthesis
BFP-C	BFP- canine-optimized
BFP-H	BFP-human-derived
CDA	cervical-diaphyseal angle
CFU	colony-forming units
CON	control group
D	diameter
EBM	electron beam melting
*E. coli*	*Escherichia coli*
H	height
HD	high-dose bacterial group
H&E	Hematoxylin and Eosin
HHA	hemi-hip arthroplasty
L	neck length
LD	low-dose bacterial group
MD	middle-dose bacterial group
MPF	maximum pull-out force
OFT	open field test
POD	postoperative days
PJI	periprosthetic joint infection
SD	standard deviation
*S. aureus*	*Staphylococcus aureus*
SEM	scanning electron microscope
UHD	ultra-high dose bacterial group
W	width

## Declaration of Generative AI and AI-assisted technologies in the writing process

During the preparation of this work the author(s) used [DeepSeek & ChatGPT] in order to [improve readability and language]. After using this tool/service, the author(s) reviewed and edited the content as needed and take(s) full responsibility for the content of the publication.

## Funding

This research was funded by National Key R&D Program of China (2024YFC3044700), Beijing Municipal Public Welfare Development and Reform Pilot Project for Medical Research Institutes (JYY2023-11), Beijing Natural Science Foundation - Changping Innovation Joint Fund Key Research Project (L244014), Henan Province 2024 “Three 100” Program for Clinician-Scientists (D20240023), Capital Medical University Incubating Program (PYZ24146), and Research Fund Project of Beijing Jishuitan Hospital (JST-HX-2023-001).

## Declaration of competing interest

All the authors declare no conflicts of interest with the contents of this article.
