# Supplementary material for: Establishment of a clinically relevant beagle model for periprosthetic joint infection with 3D-printed prostheses and multimodal evaluation
Source: J Orthop Translat. 2025 Jul 3;53:274–85. doi: 10.1016/j.jot.2025.05.007 (PMC12270800; doi:10.1016/j.jot.2025.05.007)
Supplement: Multimedia component 1 [file mmc1.docx]

**Supplementary material**


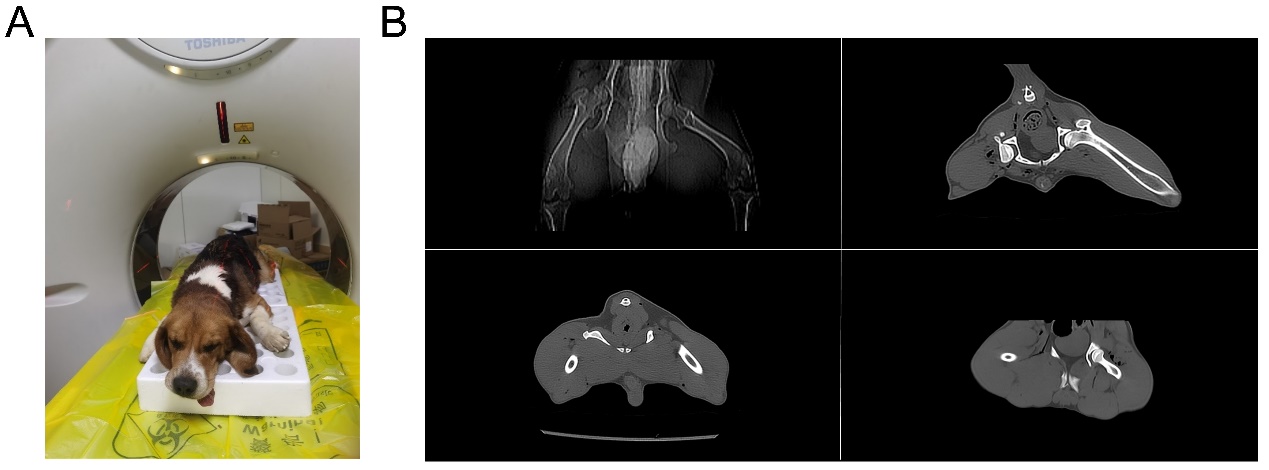


**Figure S1.** Beagle CT scan to obtain Beagle hip imaging data (**A) CT scan of a Beagle. (B) CT scan imaging data.**


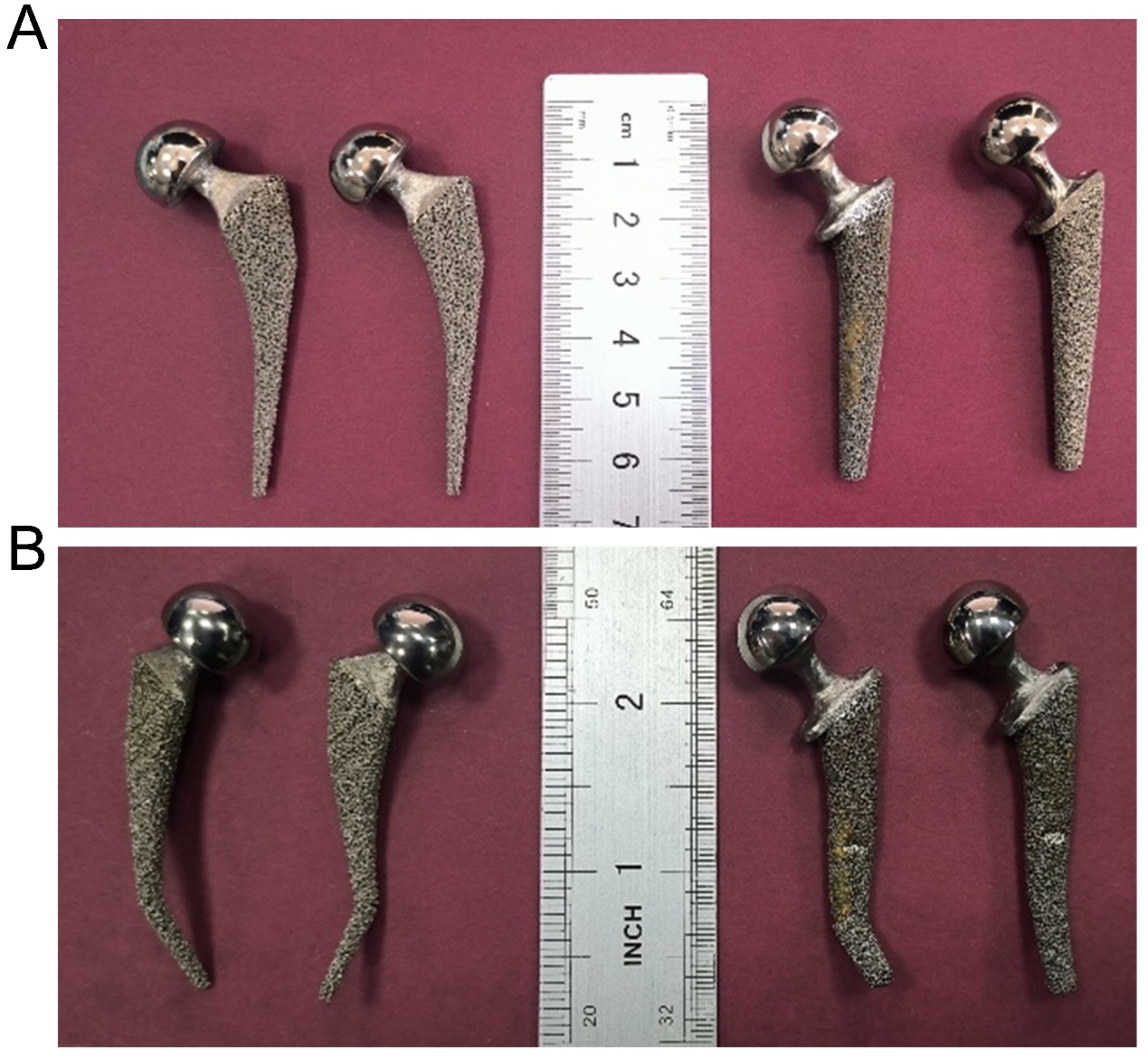


**Figure S2.** **Mechanical properties test of BFP-C and BFP-H. (A) Before mechanical test. (B) After mechanical test.**


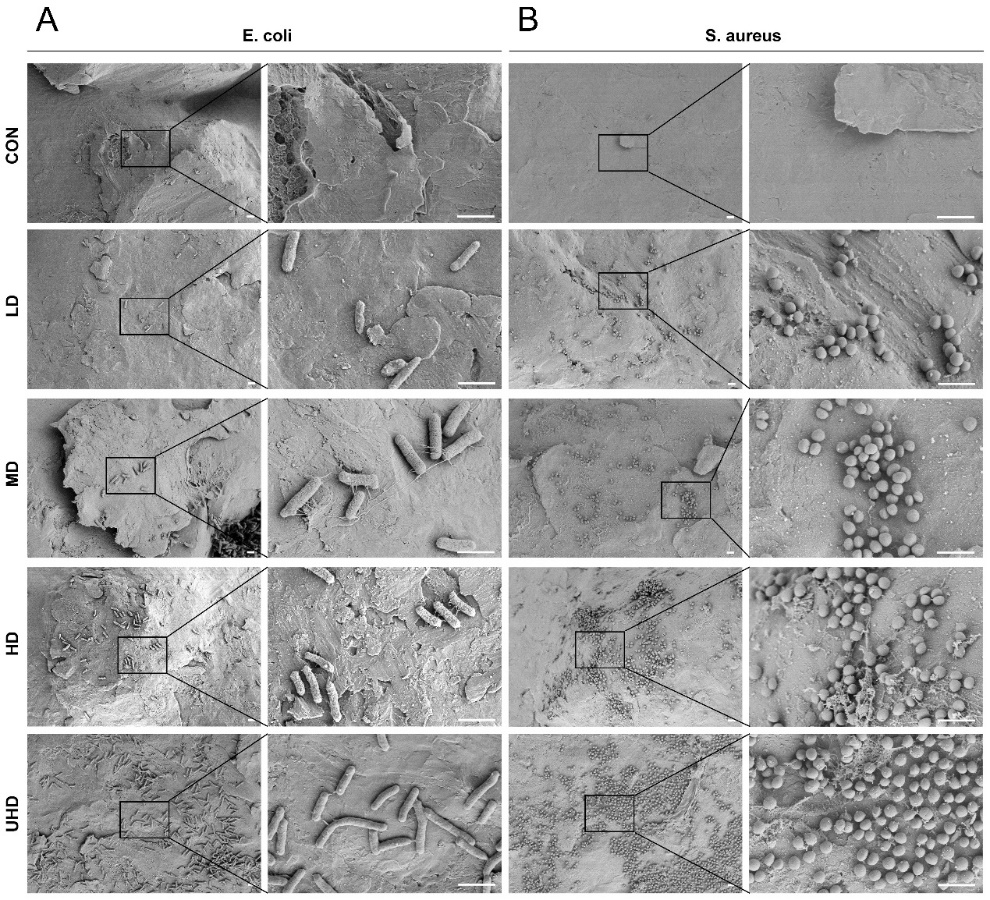


Figure S3. SEM images ‌of the two bacteria on the prosthesis. Typical aggregation forms of (A) *E. coli* and (B) *S. aureus* at CON, LD, MD, HD, and UHD concentrations. Scale bar, 100 μm.


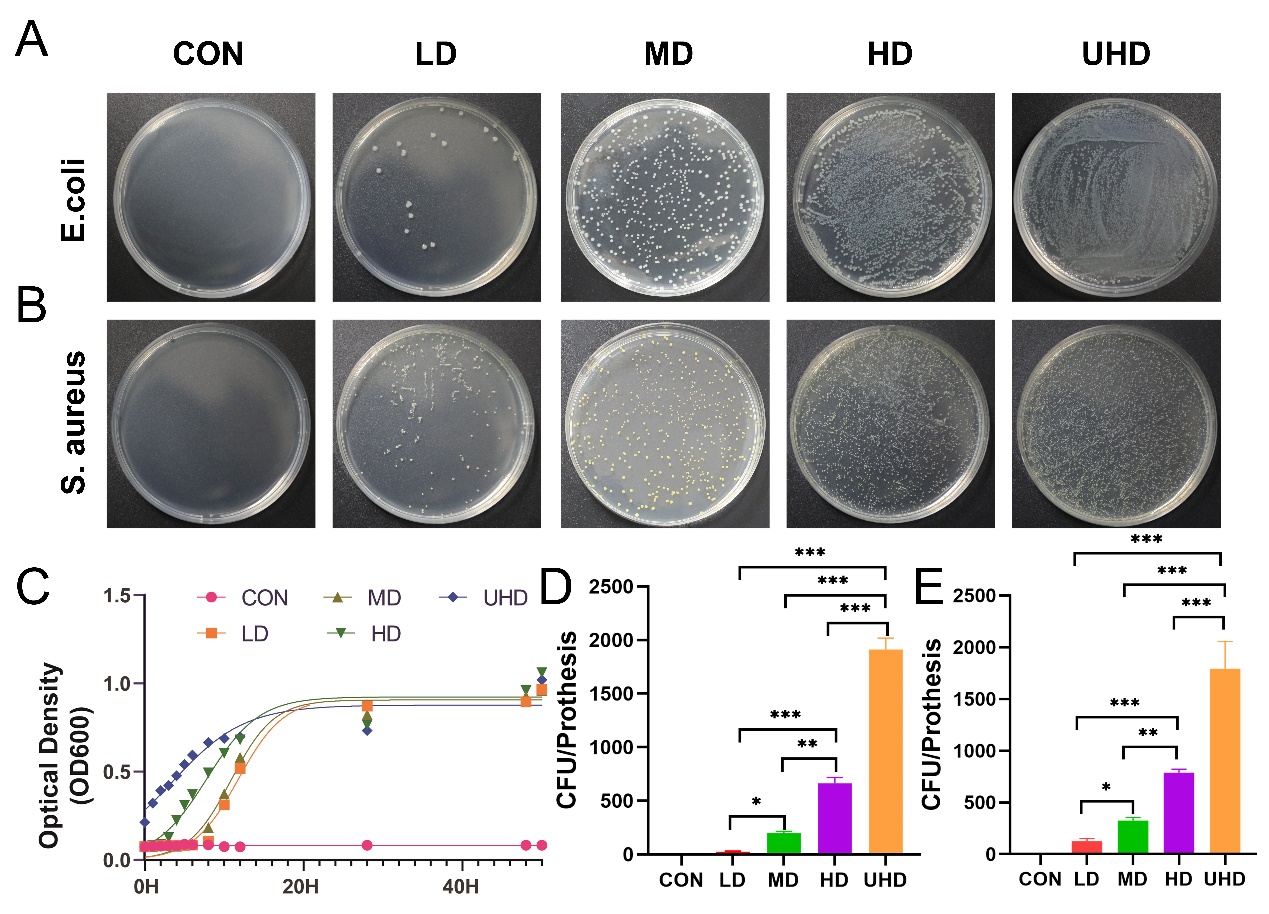


Figure S4. Proliferation identification of two kinds of bacteria on the prosthesis‌. Representative results of bacterial coated plates in (A) *E. coli* and (B) *S. aureus* at concentrations of CON, LD, MD, HD and UHD. (C) Bacterial proliferation curve. (D) The CFU statistics of *E. coli.* (E) The CFU statistics of *S. aureus*. (mean ± S.D.; **P* < 0.05, ***P* < 0.01, ****P* < 0.001; n=3).


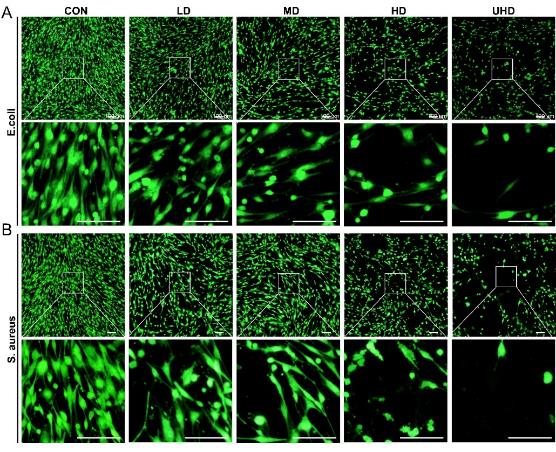


Figure S5. Image of the state of IMRCs after co-culture with two bacteria‌. The cell morphology of (A) *E. coli* and (B) *S. aureus* at concentrations of CON, LD, MD, HD and UHD. Scale bar, 100 μm.


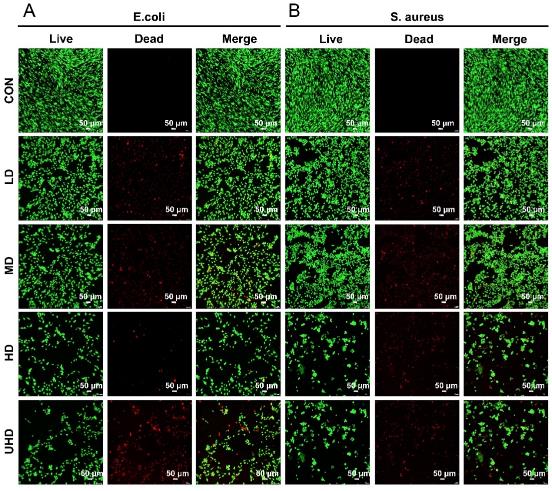


Figure S6. Viability assay of MSCs in co-culture with two bacteria ‌. The cell activities of (A) *E. coli* and (B) *S. aureus* at concentrations of CON, LD, MD, HD and UHD. Green, living cells; Red, dead cells. Scale bar, 50 μm.


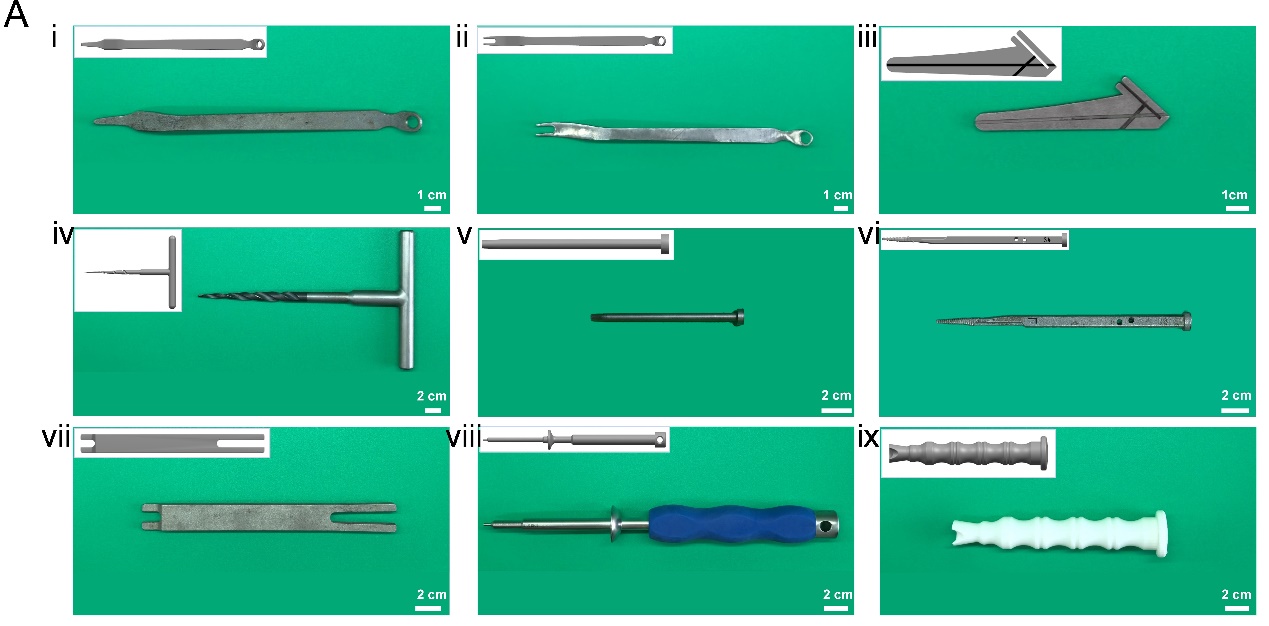


**Figure S7. Manufacture of the specialized surgical tools for Beagle HHA (A) surgical tools. i, Single-prong femoral head elevator; ii, Double-prong femoral head elevator; iii, Femoral head osteotomy guide; iv, Retractor; v, BFP angle indicator; vi, Medullary reamers; vii, Reamer punch; viii, BFHP inserter; ix, Femoral head repositioner.**


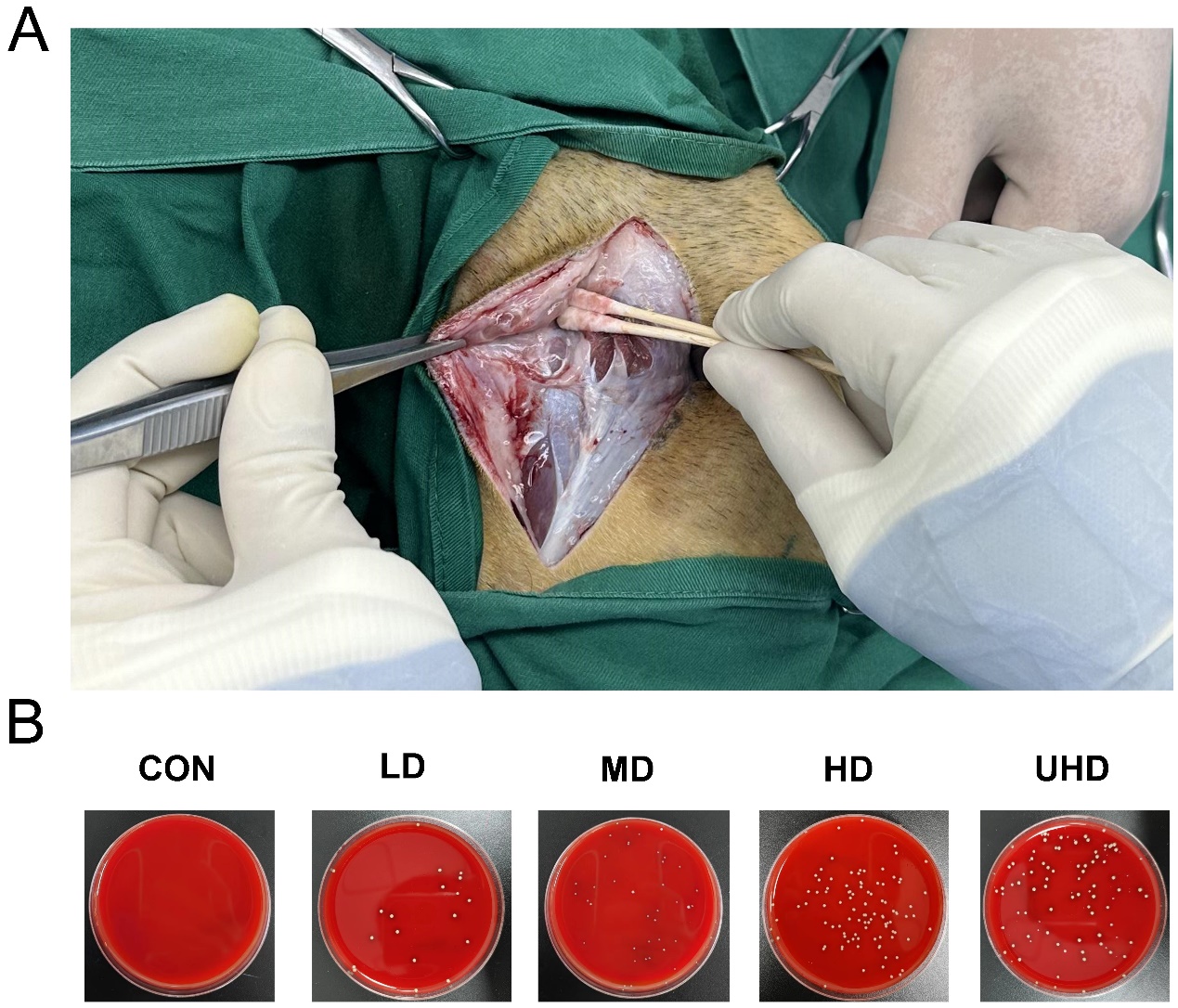


**Figure S8.** **Wound exudate culture identification to verify the absence of cross-infection with other bacteria.** **(**A) Bacterial culture performed using sterile swabs to collect subcutaneous pus. (B) Bacterial culture results showing morphology consistent with *S. aureus*, with no evidence of other bacterial infections.

<https://www2.cloud.editorialmanager.com/jotr/download.aspx?id=159523&guid=9c75c632-5805-4ca4-b823-32894a19651f&scheme=1>

**Video S1: Hip joint mobility.** On POD 28**, right hip joint mobility in the CON group Beagles showed significant recovery.**

<https://www2.cloud.editorialmanager.com/jotr/download.aspx?id=159524&guid=bff2fc1f-144d-4410-8b23-5405fb4363d3&scheme=1>

**Video S2: Wound pus extrusion.** On POD 28, the LD group showed sinus tract formation and pus exudation from the wound.

**Table S1: Radiological scoring system**

| Criterion | Score | Description |
| --- | --- | --- |
| Bone destruction | 0-3 | 0: No bone destruction observed 1: Mild bone destruction, localized to a small area 2: Moderate bone destruction, affecting a larger area 3: Severe bone destruction, extensive damage throughout the bone |
| Periosteal reaction | 0-3 | 0: No periosteal reaction observed 1: Mild periosteal reaction, slight thickening of the periosteum 2: Moderate periosteal reaction, noticeable thickening and new bone formation 3: Severe periosteal reaction, extensive new bone formation and significant |
| Prosthesis position change | 0-3 | 0 points: No change in prosthesis position 1 point: Slight displacement, minimal movement of the prosthesis 2 points: Moderate displacement, noticeable movement of the prosthesis 3 points: Severe displacement, significant movement or loosening of the prosthesis |
| Soft tissue changes around prosthesis | 0-3 | 0 points: No changes in soft tissue observed 1 point: Mild changes, slight swelling or fluid accumulation 2 points: Moderate changes, noticeable swelling and fluid accumulation 3 points: Severe changes, extensive swelling, fluid accumulation, and possible abscess formation |

Table S2: Wound scoring system

| Criterion | Description | Score | Details |
| --- | --- | --- | --- |
| Erythema (Redness) | Redness around the wound | 0-3 | 0: No erythema 1: Mild erythema 2: Moderate erythema 3: Severe erythema |
| Exudate (fluid discharge) | Fluid discharge from the wound | 0-3 | 0: No exudate 1: Mild exudate 2: Moderate exudate 3: Severe exudate |
| Healing status | Degree of wound healing | 0-3 | 0: Completely healed 1: Mildly unhealed 2: Moderately unhealed 3: Severely unhealed |

Table S3: Masson staining scoring system

| Criterion | Description | Score | Details |  |
| --- | --- | --- | --- | --- |
| Collagen deposition | Proportion of blue area (collagen fibres) | 0-3 | 0: No collagen deposition 1: Mild collagen deposition 2: Moderate collagen deposition 3: Severe collagen deposition |  |
| New bone formation | Proportion of red area (new bone formation) | 0-3 | 0: No new bone formation 1: Mild new bone formation 2: Moderate new bone formation 3: Severe new bone formation | |
| Bone matrix damage | Proportion of bone trabecular gaps | 0-3 | 0: Intact bone trabeculae 1: Mild damage 2: Moderate damage 3: Severe damage |  |
| Fibrosis degree | Thickness of collagen fibres |  | 0: No fibrosis 1: Mild fibrosis 2: Moderate fibrosis  3: Severe fibrosis |  |
